# Isolation and characterization of gut bacteria associated with the degradation of host-specific terpenoids in *Pagiophloeus tsushimanus* (Coleoptera: Curculionidae) larvae

**DOI:** 10.1093/jisesa/iead019

**Published:** 2023-04-19

**Authors:** Heng Qiao, Han Zhu, Hui Li, Hongjian Chen, Shouyin Li, Cong Chen, Dejun Hao

**Affiliations:** Co-Innovation Center for Sustainable Forestry in Southern China, Nanjing Forestry University, Nanjing 210037, China; College of Forestry, Nanjing Forestry University, Nanjing 210037, China; Co-Innovation Center for Sustainable Forestry in Southern China, Nanjing Forestry University, Nanjing 210037, China; College of Forestry, Nanjing Forestry University, Nanjing 210037, China; Co-Innovation Center for Sustainable Forestry in Southern China, Nanjing Forestry University, Nanjing 210037, China; College of Forestry, Nanjing Forestry University, Nanjing 210037, China; Co-Innovation Center for Sustainable Forestry in Southern China, Nanjing Forestry University, Nanjing 210037, China; College of Forestry, Nanjing Forestry University, Nanjing 210037, China; Co-Innovation Center for Sustainable Forestry in Southern China, Nanjing Forestry University, Nanjing 210037, China; College of Forestry, Nanjing Forestry University, Nanjing 210037, China; Co-Innovation Center for Sustainable Forestry in Southern China, Nanjing Forestry University, Nanjing 210037, China; College of Forestry, Nanjing Forestry University, Nanjing 210037, China; Co-Innovation Center for Sustainable Forestry in Southern China, Nanjing Forestry University, Nanjing 210037, China; College of Forestry, Nanjing Forestry University, Nanjing 210037, China

**Keywords:** *Pagiophloeus tsushimanus*, intestinal bacteria, plant secondary metabolite, *Cinnamomum camphora*, functional analysis

## Abstract

Insect intestinal bacteria play an important role in resisting defensive substances of host plants. *Pagiophloeus tsushimanus* (Coleoptera: Curculionidae) feeds exclusively on camphor trees (*Cinnamomum camphora*, Laurales: Lauraceae) in China, causing substantial economic and ecological losses. It is unclear how the larvae of *P. tsushimanus* outcome the main secondary metabolites of *C. camphora* such as *D*-camphor, eucalyptol, and linalool. In this study, we isolated terpenoid-degrading bacteria from the gut of *P. tsushimanus* larvae by using selective culture medium. Maximum likelihood phylogenetic analyses were performed with 16S rDNA sequences to identify the bacteria, and results showed ten strains belonged to four genera, including *Pseudomonas*, *Enterobacter*, *Serratia*, and *Corynebacterium*. Then, gas chromatography was employed to determine the degradability of *D*-camphor, eucalyptol, and linalool by the isolated strains, results showed that Z5 strain (i.e., *Corynebacterium variabile*, Actinomycetales: Corynebacteriaceae), F1 strain (i.e., *Pseudomonas aeruginosa*, Pseudomonadales: Pseudomonaceae), and A3 strain (i.e., *Serratia marcescens*, Enterobacterales: Enterobacteriaceae) had the highest degradation rates of *D*-camphor, linalool, and eucalyptol, respectively. The intestinal bacteria were capable of terpenoid degradation in vitro, which suggested that these gut bacteria associated with *P. tsushimanus* play an important role in overcoming host plant secondary metabolite defense, thereby facilitating the host specialization of this pest.

The diversity and evolutionary success of insects depends partly on their inextricable relationships with beneficial microorganisms (Engel and Moran 2013). Insects associate with many microorganisms, and in their guts there are many symbiotic bacteria that have a certain impact on the insect growth and development, food digestion, and immunity (Zhang et al. 2021). Insect microorganisms participate in upgrading nutrient-poor diets; providing essential amino acids, vitamins, and other nutrients (Moran et al. 2003); detoxifying insecticides and protecting the host from poisoning (Kikuchi et al. 2012); protecting from predators, parasites, and pathogens (Kaltenpoth and Engl 2014); contributing to inter- and intra-specific communication; and inhibiting the defense of host plants (Berasategui et al. 2017, Paniagua Voirol et al. 2018).

Host plants can produce a large class of secondary metabolites, which repel or prevent insect feeding, cause direct intoxication and growth inhibition, and eventually produce the insect death (Jamwal et al. 2018, Divekar et al. 2022). Terpenoids are the most extensive plant secondary metabolites and have direct antifeedant and toxic effects on natural enemies (Bennett and Wallsgrove 1994). For example, in previous studies, volatile terpenoids extracted from *Solanum nigrum*, such as 7-epizingiberene (i.e., sesquiterpene), have been shown to be toxic to the silver leaf whitefly *Bemisia tabaci*, thereby reducing insect feeding on plants (Rosen et al. 2015). Correspondingly, herbivorous insects have evolved different mechanisms in the long-term interaction between plants and herbivorous insects in order to overcome the noxious effects of plant defense (Hammer and Bowers 2015). The gut bacteria of some insects can play an essential role in digesting and detoxifying foods containing plant secondary metabolites (Hehemann et al. 2010, Paniagua Voirol et al. 2018). Some reports have shown that bacteria associated with the mountain pine beetle *Dendroctonus ponderosae* (i.e., *Pseudomonas* and *Rahnella*) were proven to be able to reduce concentrations of terpenes, which directly or indirectly contribute to the capacity of the mountain pine beetle *D. ponderosae* to overcome host tree defenses (Adams et al. 2013, Boone et al. 2013). Currently, knowledge of the intestinal bacteria assisting host insects in detoxifying secondary metabolites is limited to some easily manipulated model insects, such as the diamondback moth *Plutella xylostella*, cabbage root fly *Delia radicum*, coffee berry borer *Hypothenemus hampei*, and pine weevils *Hylobius abietis* (Petersson and Örlander 2003, Jaramillo et al. 2006, Dosdall et al. 2012, Xia et al. 2017).

*Pagiophloeus tsushimanus* (Coleoptera: Curculionidae) is a phloem-feeding weevil that attacks camphor trees, *Cinnamomum camphora* (Laurales: Lauraceae) (Morimoto 1982). This weevil was first reported in Shanghai in 2014 and identified as a new record species in China (Huang et al. 2014). Over the past few years, this weevil pest has rapidly reached epidemic numbers, causing widespread damage to camphor plantations in our study areas (Songjiang District, Shanghai Province, China). From late April to early June, males and females mating often occurs on the sunny side of the crown, and it is observed that adults repeatedly mate and lay eggs. After mating, females crawl along the trunk below the canopy to find a suitable oviposition site and form an egg chamber in the cambium, and all females only lay one egg at a time, with one in each chamber. *Pagiophloeus tsushimanus* has one life cycle per year in Shanghai. Adults and 3–5 instar larvae overwinter from early November to early April. Adults overwinter in grooves on the underside of branches or branch nodes, and larvae overwinter in tunnels. Adults feed on the bark of twigs, and occasionally on new buds. Larvae (1st–2nd instar) feed on the phloem, while 3rd–5th instar can bore into the phloem and the cambium (Chen et al. 2021). Therefore, the larvae were supposed to have the ability to overcome toxic secondary metabolites produced by camphor trees when feeding on the phloem. We previously analyzed the phloem and cambium of the trunk at the feeding site of *P. tsushimanus* larvae by gas chromatography–mass spectrometry (GC–MS) and found 19 main secondary metabolites. Among them, the relative content of the top three substances were eucalyptol (31.89%), *D*-camphor (19.22%), and linalool (10.16%) (Li et al. 2022). Studies have shown that the secondary metabolites of *C. camphora* are considered to have a high degree of resistance to a variety of herbivorous insects (Jiang et al. 2016, Wu et al. 2020). In our previous study, however, low doses of exogenous camphor can instead promote growth and development of *P. tsushimanus* (Li et al. 2023), suggesting that this specialist weevil appears to have evolved a strategy adapted to its host-specific chemical defenses. Thus, this deserves in-depth studies to investigate whether the symbiotic gut bacteria in *P. tsushimanus* larvae play an important role in detoxifying and metabolizing these hosts’ toxins.

In this study, we isolated the gut bacteria of *P. tsushimanus* larvae and identified the strains by combining morphological characteristics as well as phylogenetic analysis for the first time. We further tested the terpenoids degradability of isolated strains by GC measurements. Our results may further elucidate interactions between *P. tsushimanus* and its gut bacteria in terms of host plant adaptations.

## Materials and Methods

### Insect Collection and Dissection

*Pagiophloeus tsushimanus* larvae were collected from a camphor plantation in Songjiang District, Shanghai, China. The larvae were subjected to starvation in an incubator at a temperature of 28 ± 0.5 °C and a relative humidity of 70 ± 5% in the dark for 24 h. Thirty 4th-instar larvae were randomly selected, and the surface of the larvae was disinfected with 70% alcohol and soaked in phosphate buffer solution (PBS), which was repeated three times to avoid external contamination (Morales-Jiménez et al. 2012). After dissection under sterile conditions, the guts were immediately transferred to a 2 ml sterile centrifuge tube with 0.5 ml of PBS for subsequent grinding.

### Isolation and Purification of Terpenoid-Degrading Bacteria

Eucalyptol [99.5% purity, Chemical Abstracts Service (CAS) number: 470-82-6] (Aladdin, China), *D*-camphor (96% purity, CAS number: 464-49-3) (Aladdin, China), and linalool (98% purity, CAS number: 78-70-6) (Aladdin, China) were added to 50 ml of Bushnell Haas broth liquid medium at a ratio of 1% (w/v), and the liquid BHB medium consisted of 0.20 g/l MgSO_4_, 0.02 g/l CaCl_2_, 1.0 g/l KH_2_PO_4_, 1.0 g/l K_2_HPO_4_, 1.0 g/l (NH_4_)_2_SO_4_, 0.05 g/l FeCl_3_ (pH 7.0–7.2). Then, the larval intestinal abrasive was inoculated into the medium at a ratio of 3% (v/v), without intestinal abrasive medium as a control. After inoculation, the medium was incubated at 28 °C and 150 rpm. When the bacterial liquid was cloudy, the culture was serially diluted from 10^−1^ to 10^−6^, and 100 μl of dilution was coated on LB, NA, and TYA solid media, with three replicates per medium. The LB, NA, and TYA solid media consisted of 10 g/l peptone, 5 g/l yeast extract, 10 g/l NaCl, and 18 g/l agar (pH 7.2); 10 g/l peptone, 3 g/l beef extract, 5 g/l NaCl, and 18 g/l agar (pH 7.2); and 2.0 g/l yeast extract, 2.0 g/l beef paste, 6.0 g/l peptone, 40 g/l glucose, 3.0 g/l ammonium acetate, 0.5 g/l KH_2_PO_4_, 0.2 g/l neutral red, 0.2 g/l MgSO_4_·7H_2_O, 0.02 g/l FeSO_4_·7H_2_O, and 15 g/l agar (pH 6.5), respectively. After coating, the Petri dishes were incubated at 28 °C for 48 h in a bacteriological incubator. After selecting single colonies, the isolated and purified strains were preserved in 25% glycerol at −80 °C.

### Morphological Identification of Terpenoid-Degrading Bacteria

Referring to Shen and Chen (2018), morphological characteristics such as color, size, shape, edges, bulges, transparency, and gloss of individual colonies were observed and described. And the morphology of the strain was observed by Gram staining, spore, capsule, and flagella staining.

### Molecular Identification of Terpenoid-Degrading Bacteria

The purified bacterial strains were inoculated in LB liquid medium. After inoculation, these media were incubated at 28 °C, with shaking at 220 rpm for 12 h, and 1 ml of the cloudy bacterial solution was taken. Then, the bacterial genomic DNA was extracted by the E.Z.N.A.Soil DNA Kit (Omega Bio-Tek, USA) following the manufacturer’s instructions.

PCR amplification was performed using the extracted total bacterial DNA as a template with Taq DNA polymerase (Accurate, China) and 16S rDNA universal primers 27F (5ʹ-AGAGTTTGATCCTGG CTCAG-3ʹ) and 1492R (5ʹ-GGTTACCTTGTTACGACTT-3ʹ). The PCR reaction volume was 25 μl: 9.5 μl ddH_2_O, 12.5 μl 2 × Accurate Taq Master Mix, 1.0 μl gDNA (50 ng/μl), 1 μl primer 27F (10 μmol/l), and 1 μl primer 1492R (10 μmol/l). The PCR program included 30 s at 94 °C; 30 cycles of 98 °C for 10 s, 55 °C for 30 s, 72 °C for 2 min; and 72 °C for 2 min. Then, the PCR products were separated on a 1% agarose gel and sequenced by Jie Li Biology Co. Ltd (Shanghai, China).

The sequences of different bacterial isolates were blasted by online NCBI Blastn program (http://www.ncbi.nih.gov/blast). Multiple sequence alignments of 16S rDNA gene sequences of terpenoids-degrading bacteria and their closest relatives or members were performed by MAFFT version 7 software (https://www.ebi.ac.uk/Tools/msa/mafft/). Phylogenetic analyses were conducted using Bayesian inference (BI) and Maximum likelihood (ML). ModelFinder (Kalyaanamoorthy et al. 2017) was used to select the best-fit model using AIC criterion in PhyloSuite (Zhang et al. 2020). Best-fit model according to AIC: TIM3+F+R3 for BI and ML. ML analyses were conducted with IQ-TREE (Nguyen et al. 2015) using aligned sequences. Clade support was assessed by 5,000 bootstrap pseudoreplicates with the standard bootstrap of 1,000 replicates of the aligned data set. Bayesian inference (BI) analyses were analyzed in MrBayes v. 3.2.6 (Ronquist et al. 2012). Two million generations were run, with 25% of the generations as burn-in. The potential scale reduction factor (PSRF) close to 1.0 and the average standard deviation of split frequencies below 0.01 were accepted.

The DNA sequences of the A1, A2, A3, F1, F2, Z1, Z2, Z3, Z4, and Z5 strains were uploaded to GenBank with the numbers of ON153202, ON153203, ON153204, ON153205, ON153206, ON153207, ON153208, ON153209, ON153210, and ON153211, respectively.

### Degradation Capacity of Bacterial Isolations

#### Sample collection and processing for GC.

The purified and preserved strain was inoculated in 30 ml of LB liquid medium for activation. The activated bacterial liquid was centrifuged at 10,000 rpm for 10 min at room temperature. The medium was discarded, and the bacteria were retained. Then, the bacteria were continuously washed with sterile water three times, and the concentration of bacterial liquid was diluted to an OD600 value of 1.5. After activation, 100 μl bacterial liquid was inoculated into 3 ml BHB medium containing 1% (w/v) eucalyptol, and the BHB medium without bacterial liquid was used as the blank control group. After shaking culture at 28 °C and 150 r/min for 10 days, 2 ml of chromatographically pure n-hexane (98% purity, CAS number: 110-54-3) (Aladdin, China) was added, treated in a sealed shaker for 3 h, placed upright, and treated overnight at −20 °C. After thawing at room temperature, 1 ml of hexane extract was transferred into a 2 ml autosampler vial (Figure S1). There were five replicates in the experimental group and the control group.

#### Sample processing.

Ten microlitres of n-hexane solution with 50% isobutylbenzene (99.5% purity, CAS number: 538-93-2) (Aladdin, China) was added as the internal standard (IS) for each sample, GC was used for quantitative analysis, and the sample was stored in a −20 °C refrigerator before analysis. Standard treatment: eucalyptol, D-camphor, and linalool standards with different masses were added to the n-hexane solution. Then, 10 μl n-hexane solution with 50% isobutylbenzene was added as the IS to prepare the standard with mass ratios of 1:1, 2:1, 3:1, 4:1, and 5:1. After mixing evenly, the standard was placed in a refrigerator at −20 °C to be tested.

#### Gas chromatography.

The degradation capacity of each strain for D-camphor, eucalyptol, and linalool was determined by a GC system (6890N, Agilent Technologies, USA) equipped with a flame ionization detector with an ionization energy of 70 eV and a fused silica capillary column (DB-5, 30 m × 0.32 mm × 0.25 μm, Agilent J & W GC columns, USA). The carrier gas was nitrogen, with a flow rate of 1 ml/min. The injector and mass transfer line temperatures were both 250 °C. The injection volume was 1 μl, with a hydrogen flow of 40 ml/min, air flow of 450 ml/min, and nitrogen flow of 15 ml/min. The starting temperature of the heating program was kept at 40 °C for 1 min, 20 °C/min to 110 °C for 1 min, 4 °C/min to 120 °C for 1 min, and 20 °C/min to 200 °C. The total running time was 13 min.

*Calculation of the Degradation Rate.* A 1 μl standard sample was taken for GC analysis. The peak retention time of each component in the sample to be measured is compared with that in the standard sample to determine the component to be tested. The standard samples with different mass ratios were determined by GC. According to the peak area ratio of standard samples to IS, the standard curve fitting was plotted by Origin 2021 (OriginLab, UK) to obtain the linear regression equation. The quantifications were carried out using the internal standard method (Deng and Zhang 2019), the formula of the relative correction factor f is as follows:


$$\begin{array}{l} f=\frac{As}{ms}/\frac{Ar}{mr}\; \end{array}$$

*As*: peak area of internal standard sample; *Ar*: peak area of standard sample; *ms*: internal standard mass; *mr*: standard sample mass.

According to the peak area (*Ai*) of the tested component, the peak area ratio of the internal standard, and the relative correction factor, the mass (*mi*) of the tested component was calculated as follows:


$$\begin{array}{l} mi=f\times \left(Ai/\frac{As}{ms}\;\right) \end{array}$$

Finally, the degradation rate of the strain (*D*_*E*_) was calculated according to the following formula:


$$\begin{array}{l} DE\left(\%\right)=\left(miC-miE\right)/miC\; \end{array}$$

*mi*_*C*_: the mass of tested components in the control group; *mi*_*E*_: the mass of tested components in the experimental group.

Differences in the degradation rates of *D*-camphor and eucalyptol by different *D*-camphor degrading strains and eucalyptol-degrading strains were determined using one-way analysis of variance (ANOVA), followed by Tukey’s test (*P* < 0.05). Difference in the linalool degradation rate between F1 strain and F2 strain was determined by independent sample *t*-test (*P* < 0.05). All statistical analyses were performed using SPSS 22.0 (IBM SPSS Statistics, USA) and plotted with Origin 2021 (OriginLab, UK).

## Results

### Isolation and Morphological Characterization of Terpenoid-Degrading Bacteria in the Gut of *P. tsushimanus* Larvae

**Fig. 1.**
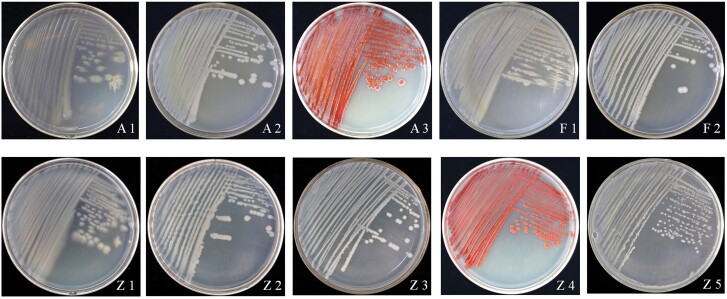
The terpenoid-degrading bacteria in the gut of *P. tsushimanus* larvae (A1, A2, A3, F1, F2, Z1, Z2, Z3, Z4, and Z5). *D*-camphor-degrading bacteria: Z1, Z2, Z3, Z4, and Z5; eucalyptol-degrading bacteria: A1, A2, and A3; and linalool-degrading bacteria: F1 and F2.

A total of ten strains of bacteria were isolated from the intestines of 4th-instar larvae. Three eucalyptol-degrading bacteria were coded as A1, A2, and A3; two linalool-degrading bacteria were coded as F1 and F2; and four *D*-camphor-degrading bacteria were coded as Z1, Z2, Z3, and Z4 (Fig. 1). The morphological characteristics of the ten strains are shown in Table 1. The bacterial isolates exhibited diverse colony sizes, colors, margins, texture, and so on. The sizes of six isolates (i.e., A1, A2, A3, F1, Z1 and Z2) were medium, and four isolates (i.e., Z2, Z3, Z4, and Z5) were small. The colors of A2, F2, and Z3 were milky; A1, F1, Z1, Z2, and Z5 were yellowish; and A3 and Z4 were red. The margins of A2, F2, Z2, and Z3 were neat; A1, F1, and Z1 were irregular margins; and A3, Z4, and Z5 were regular margins. The shapes of the ten isolates were rod-like, and none had spores; four isolates (i.e., A1, F1, Z1, and Z2) had capsulatum, and nine isolates had flagellum. Regarding Gram staining, only the strain F5 was Gram positive, and the remaining isolates were Gram negative.

**Table 1.** Morphological characteristics of terpenoid-degrading bacteria in the gut of *P. tsushimanus larvae*

Strain	Bacterium shape	Gram staining	Spores	Capsule	Flagella	Colony morphology
A1	Rod	Gram −ve	–	+	+	Yellowish, medium-size, round, irregular margin, opaque, smooth
A2	Rod	Gram −ve	–	–	+	Milky, medium-size, round, neat margin, convex, opaque, smooth
A3	Rod	Gram −ve	–	–	+	Red, medium-size, round, regular margin, convex, opaque, smooth
F1	Rod	Gram −ve	–	+	+	Yellowish, medium-size, round, irregular margin, opaque, smooth
F2	rod	Gram −ve	–	–	+	Milky, medium-size, round, neat margin, convex, opaque, smooth
Z1	rod	Gram −ve	–	+	+	Yellowish, medium-size, round, irregular margin, opaque, smooth
Z2	rod	Gram −ve	–	+	+	Yellowish, small-size, round, neat margin, convex, opaque, sticky
Z3	rod	Gram −ve	–	–	+	Milky, small-size, round, neatly margin, convex, opaque, smooth
Z4	rod	Gram −ve	–	–	+	Red, small-size, round, regular margin, convex, opaque, smooth
Z5	rod	Gram +ve	–	–	–	Yellowish, small-size, round, regular margin, convex, translucent, smooth

### Molecular Identification of Terpenoid-Degrading Bacteria in the Gut of *P. tsushimanus* Larvae

It was found that the identity between the sequences of 10 strains and the known 16S rDNA sequences in the database ranged from 99.57% to 100% by BLAST. As shown in Table 2, the BLAST results showed that the eucalyptol-degrading strains A1, A2, and A3 had the highest identity with *Pseudomonas aeruginosa*, *Enterobacter* sp., and *Serratia marcescens*, respectively; the linalool-degrading strains F1 and F2 had the highest identity with *P. aeruginosa* and *Enterobacter* sp., respectively; and the *D*-camphor-degrading strains Z1, Z2, Z3, Z4, and Z5 had the highest identity with *P.* aeruginosa, *Pseudomonas* sp., *Enterobacter* sp., *S. marcescens*, and *Corynebacterium variabile*, respectively.

**Table 2.** 16S rDNA identification results of degrading bacteria in larval gut of *P. tsushimanus*

Strain	Strain with the highest identity in Blast	GenBank accession number	Identity (%)
A1	*Pseudomonas aeruginosa*	MF872727.1	100.00
F1	*Pseudomonas aeruginosa*	OM865894.1	100.00
Z1	*Pseudomonas aeruginosa*	KF483133.1	99.93
Z2	*Pseudomonas* sp.	AY456708.1	99.86
A3	*Serratia marcescens*	AY043386.1	99.79
Z4	*Serratia marcescens*	AY043386.1	99.72
A2	*Enterobacter* sp.	JQ765425.1	99.72
F2	*Enterobacter* sp.	JQ765425.1	99.72
Z3	*Enterobacter* sp.	JQ765425.1	99.72
Z5	*Corynebacterium variabile*	MT573863.1	99.57

**Fig. 2.**
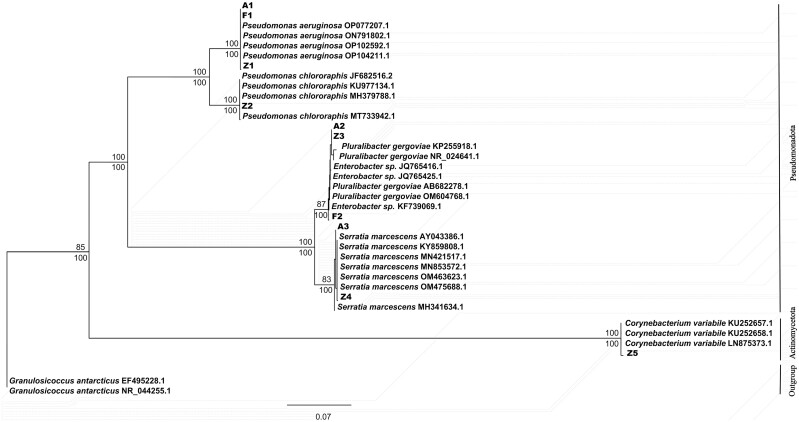
Phylogenetic analysis of the terpenoid-degrading strains inferred using 16S rDNA gene sequences. The phylogram was constructed using maximum likelihood by 5,000 bootstrap pseudoreplicates with the standard bootstrap of 1,000 replicates. ML (bootstrap values) and BI (posterior probabilities) support values ≥80% are reported above and below nodes, respectively. *Granulosicoccus antarcticus* (EF495228.1) and *G. antarcticus* (NR_044255.1) were included as outgroups.

The BI and ML analyses based on 16S rDNA gene sequences of terpenoids-degrading bacteria produced very similar tree topologies. The topology of the ML phylogram was shown as Fig. 2. All strains respectively clustered in two major clades, including Actinomycetota and Pseudomonadota clades. Among them, Z5 clustered together with the strains of *C. variabile*, which belonged to Actinomycetota; and other strains all clustered together with the strains of Pseudomonadota. To be specific, the phylogenetic tree showed that A1, F1, and Z1 clustered together with the strains of *P. aeruginosa*; Z2 clustered together with the strains of *P. chlororaphis*, and A1, F1, Z1, and Z2 both belonged to the genus *Pseudomonas*; A2, F2, and Z3 all clustered together with the strains of the genus *Enterobacter*, and A2 and Z3 both clustered together with the strains of *Pluralibacter gergovlae*; A3 and Z4 both clustered together with the strains of *S*. *marcescens*.

The above molecular identification results showed that A1, F1, and Z1 are the same species, which belonged to *P. aeruginosa*; Z2 belonged to *P. chlororaphis*; A2, F2, and Z3 all belong to *Enterobacter* sp.; A3 and Z4 belonged to *S. marcescens*; Z5 belonged to *C. variabile*. Although A1, F1, and Z1 showed little difference in morphological and molecular phylogenetic traits, we initially experimented with them as different strains due to the lack of in-depth analysis, which does not exclude the possibility that they are consistent. Likewise, A2, F2, and Z3 are also that.

### Analysis of the Degradation Capacity of Terpenoid-Degrading Bacteria in the Gut of *P. tsushimanus* Larvae

**Fig. 3.**
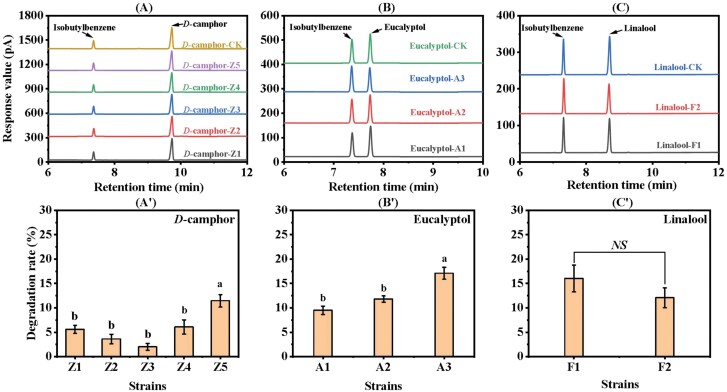
Degradation capacity of terpenoid-degrading bacteria in the gut of *P. tsushimanus* larvae. (A, Aʹ) The chromatographic diagram and degradation rate of *D*-camphor-degrading bacteria; significance was determined by Tukey’s one-way-ANOVA (*P* < 0.05); the different letters indicate significant differences, and the same letters indicate no differences. (B, Bʹ) The chromatographic diagram and degradation rate of eucalyptol-degrading bacteria; significance was determined by Tukey’s one-way-ANOVA (*P* < 0.05); the different letters indicate significant differences, and the same letters indicate no differences. (C, Cʹ) The chromatographic diagram and degradation rate of linalool-degrading bacteria; Significance was determined by independent sample *t* test (*P* < 0.05); *NS* indicates nonsignificant. All bars and error bars represent mean and standard error.

The terpenoid-degrading bacteria were added to BHB inorganic salt medium containing *D*-camphor, eucalyptol, and linalool, respectively, and then the residues of *D*-camphor, eucalyptol, and linalool in BHB were determined by GC, which could determine the degradation rates of *D*-camphor, eucalyptol, and linalool by each bacterium. The standard curve for calculating the degradation rate is shown in Figure S2. The results showed that the retention time of the absorption peaks of *D*-camphor, eucalyptol, and linalool in the chromatograms of the samples supplemented with the ten strains was consistent with that of the control chromatograms, but the content of substances as determined from the absorption peaks area was significantly reduced. For example, the retention time of the *D*-camphor absorption peak was 9.7 min, the response values of Z1, Z2, Z3, Z4, and Z5 decreased to different degrees compared with that of the control samples (Fig. 3A). Among the five *D*-camphor-degrading bacteria (Z1-5), the degradation rate of Z5 was the highest (11.44 ± 2.88%), which was significantly different from that of the other four bacteria (*F* = 10.709; df = 4, 20; *P* < 0.001); the degradation rates of Z4, Z1, and Z2 were 6.07 ± 3.27%, 5.59 ± 1.89%, and 3.61 ± 2.19%, respectively. The degradation rate of Z3 was the lowest, which was 2.00 ± 1.59% (Fig. 3Aʹ). When the retention time of the eucalyptol absorption peak was 7.7 min, and the response value of A1, A2, and A3 all decreased to different degrees, compared with that of the control group (Fig. 3B). Among the three strains of eucalyptol-degrading bacteria (A1-3), the degradation rate of A3 was 17.08 ± 2.74%, which was significantly higher than that of A1 and A2 (*F* = 16.996; df = 2, 12; *P* < 0.001). Namely, the degradation rates of A1 and A2 were 9.49 ± 1.94% and 11.80 ± 1.45%, respectively (Fig. 3Bʹ). As shown in Fig. 3C, the retention time of the linalool absorption peak was 8.7 min, and the response values of F1 and F2 both decreased to different degree compared with that of the control samples. The degradation rates of F1 and F2 were 16.03 ± 2.75% and 12.05 ± 2.04%, respectively, but there was no significant difference in the degradation rates of the two strains of linalool-degrading bacteria (*t* = 2.594; *P* = 0.780) (Fig. 3Cʹ).

## Discussion

Herbivorous insects mitigate the effects of toxic plant secondary metabolites by seeking help from symbiotic gut microbes to adapt to plant secondary metabolites (Afroz et al. 2021). The fact that symbiotic bacteria of some weevils can help host to overcome plant secondary metabolites has been confirmed (Adams et al. 2013, Boone et al. 2013). *Pagiophloeus tsushimanus* is an emerging insect pest exclusively infesting camphor trees. Its outbreak is largely due to its evolution of a high degree of adaptability to the chemical defense of specific host plants (i.e., camphor trees). In this context, clarifying the biological metabolic function of microbial symbioses in *P. tsushimanus* is not only helpful to understand the multiple interactions of insect–plant–microbes, but also provides a new insight for the comprehensive control of this pest. So, it’s of great interest to explore the microbial symbionts of *P. tsushimanus* that contribute to the detoxification of plant secondary metabolites. In this study, we first isolated and identified terpenoid-degrading bacteria in the gut of *P. tsushimanus* larvae. These bacteria were proven to degrade terpenoids when cultured separately on medium containing one terpenoid (i.e., eucalyptol, linalool, or *D*-camphor). Although A1, F1, and Z1 strain have almost no differences in morphology (including size, color, margin, shape, and so on) and molecular phylogenetic traits, but since there is no in-depth analysis, we initially treated them as different strains for the GC experiments, which does not rule out the possibility that they are consistent. Similarly, A2, F2, and Z3 are also that. After determining the terpenoid degradation rate of each bacterial strain by GC, we found that each strain could efficiently degrade on terpenoids (i.e., eucalyptol, linalool, and *D*-camphor), and that the degradation efficiencies of *Pseudomonas* and *Serratia* were relatively significant. In summary, these results revealed that the intestinal bacteria of *P. tsushimanus* have the capacity in detoxifying the host’s defensive substances and sequentially help *P. tsushimanus* adapt to host plants.

The fact that monoterpenes are utilized as energy and carbon sources by several aerobic microorganisms has been known since the 1960s (Lüddeke et al. 2012). Most reports dealt with *Pseudomonas* and *Enterobacter* species, for example *P. aeruginosa* (Förster-Fromme et al. 2006), *P. putida* (Tudroszen et al. 1977), *P. delhiensis* (Prakash et al. 2007), *E. cowanii* (Yang et al. 2007), and so on. In this research, our findings are similar to the previous studies. The bacterial identification showed that most monoterpenes-degradating strains belonged to *Pseudomonas* and *Enterobacter* species, for example, *P. aeruginosa*, *P. chlororaphis*, and *Enterobacter* sp., and *S. marcescens* and *C. variabile* also metabolize these terpenoids.

Some intestinal bacteria can help host insects defend against toxic secondary metabolites of plants and play an important role in the co-evolution of phytophagous insects and host plants (Engel and Moran 2013). In particular, symbiotic bacteria have been previously demonstrated to break down terpenoids, including sesquiterpenes, monoterpenes, and diterpenes. For example, *Pseudomonas* spp. and *Rahnella aquatilis* associated with *D. ponderosae* are capable of α-pinene degradation (Mason et al. 2014). The genera *Bacillus*, *Burkholderia*, *Enterobacter*, *Klebsiella*, and *Pseudomonas* that were isolated from leaf-cutter ants could degrade β-pinene, β-caryophyllene, or linalool (Francoeur et al. 2020). *Pseudomonas* and *Brevundimonas* isolated from the gut of bark beetles *D. ponderosae* and *D. valens* were found to degrade monoterpenes and diterpenes (Boone et al. 2013, Xu et al. 2016). *Pseudomonas abietaniphila* and *Burkholderia xenovorans* also degrade diterpenes, and they are able to utilize diterpenes as their sole carbon source (Martin and Mohn 2000, Smith et al. 2007). The intestinal bacteria of *P. tsushimanus* larvae in this study were also capable of utilizing monoterpenes as the sole carbon source. Despite the widespread ability of bacteria to degrade diterpenes as described in the literature, our contribution only test the monoterpene degradation (i.e., *D*-camphor, eucalyptol, and linalool) capacity of *P. tsushimanus* gut bacteria and have not yet been tested with other terpenoids, such as sesquiterpenes, and diterpenes.

Certainly, the degradation of plant secondary metabolites by intestinal symbiotic bacteria is not limited to terpenes (Berasategui et al. 2016). For instance, a strain of bacteria isolated from the foregut of *Spodoptera exigua* larvae can produce N-acyl amino acid hydrolases, which helps to decompose these defense-induced substances, thereby reducing the toxic effects of secondary metabolites on larvae (Ping et al. 2007). *Acinetobacter* in the gut of *L. dispar* larvae can degrade phenolic glycosides secreted by *Populus tremuloides* to protect the host from toxicity (Mason et al. 2016). *Klebsiella* and *Corynebacterium* in the gut of *Brithys crini* can degrade alkaloids in food, and *Nocardioides*, *Gordonia*, and *Curtobacterium* in the gut of *Hyles euphorbiae* can tolerate latex secreted by plants and protect the gut of insects (Vilanova et al. 2016). Likewise, intestinal bacteria that degrade plant secondary metabolites also exist in other organisms. Gut microbes of the desert woodrat *Neotoma lepida* degrade phenolic compounds produced by the toxic creosote bush *Larrea tridentata* (Kohl et al. 2014, 2016). In addition to bacteria, symbiotic fungi can also degrade plant secondary metabolites for hosts. For example, the yeast-like symbiont of the *Lasioderma serricome* has 1-naphthyl acetate esterase activity, which can metabolize exogenous toxic substances (Shen and Dowd 1991).

Interestingly, some intestinal microorganisms can detoxify and reutilize toxic plant secondary metabolites produced by host plants. The terpenes degraded by intestinal microorganisms might be reused by insects (DiGuistini et al. 2011). For instance, the members of the gut community of *D. ponderosae* (i.e., *Rahnella* sp., *Pantoea* sp., and *Stenotrophomonas* sp.) and *D. rhyzophagus* (i.e., *Pseudomonas* sp., *Rahnella* sp., and *Klebsiella* sp.) can fix nitrogen and provide nutrition (Morales-Jiménez et al. 2012). Our results showed that the absorption peaks of these secondary metabolites were significantly reduced and no new peaks were detected in these chromatograms. Therefore, we suspected that these gut bacteria can not only degrade *D*-camphor, eucalyptol, and linalool, but also reutilize decomposition products for their own growth. However, other researchers speculated that no breakdown products were detected via GC, which could be a result of complete degradation of the compounds into components of central metabolism (Marmulla and Harder 2014). Therefore, these results need to be further verified. Another interesting thing is that, the same or taxonomically related bacteria may play different roles in different insect systems and different microenvironments. In the paper, the detected bacteria included *S. marcescens* and *Pseudomonas* were considered as highly efficient terpene degrading bacteria. However, in many insect systems, some *S. marcescens* and *Pseudomonas* show extremely high insect pathogenicity. For instance, *S. marcescens* strain SRM and EML-SE1 showed high pathogenicity to *Helicoverpa armigera* and *P. xylostella* larvae, respectively (Jeong et al. 2010, Mohan et al. 2011). *Pseudomonas fluorescens* CHA0 and *P. chlororaphis* PCL1391 could kill insects via oral infection (Ruffner et al. 2013). We speculate that these strains may produce different proteins, including enzymes, toxin proteins, etc.

Bacterial communities are multifunctional, with multiple members performing different roles within the same species, such as bacterial communities associated with bark beetles (Six 2013). The degradation rates of eucalyptol, *D*-camphor, and linalool showed that the bacterial communities associated with *P. tsushimanus* had different degradation efficiencies in degrading terpenoids. Among the ten isolated terpenoid degradation strains, *P. aeruginosa* and *Enterobacter* sp. are effective in degradation of eucalyptol, *D*-camphor, and linalool, but *C. variabile* had the highest degradation rate of *D*-camphor, and the rate of linalool degradation by *P. aeruginosa* was the highest. *S. marcescens* had the highest decomposition rate of eucalyptol. This was consistent with the findings of other insect gut bacteria, such as *Pseudomonas* sp. and *Serratia* sp. of *D. valens*, which perform main roles in degrading α-pinene and tolerating high levels of α-pinene in vitro, and helping other microorganisms and hosts overcome the α-pinene defense of pine trees (Xu et al. 2016). Thus, it can be seen that each genus of bacteria plays different roles in detoxification. However, we only tested the degradation of *D*-camphor, eucalyptol, and linalool by intestinal bacteria in vitro by GC. It was also necessary to perform re-infected bioassays to a further evaluation of the biological activity of isolated monoterpene degradation strains to *P. tsushimanus* and verify the metabolic activities of plant secondary metabolites in vivo.

In conclusion, similar to other herbivorous insects, the intestinal microbiota of *P. tsushimanus* is obviously important for determining palatable plant substrates. These bacteria could degrade secondary metabolites of *C. camphora* to various extents, especially *Pseudomonas aeruginosa* with the ability to degrade these three secondary metabolites (i.e., *D*-camphor, eucalyptol, and linalool), which highlighted the important role of intestinal bacteria in degrading secondary metabolites of *C. camphora*. It must be noted that our current work is not sufficient, and in-depth studies should focus on testing the physiological role in vivo and clarifying the metabolic pathways of these bacteria in degrading secondary metabolites. 

## Supplementary Material

iead019_suppl_Supplementary_Material

## Data Availability

The gene sequences of the A1, A2, A3, F1, F2, Z1, Z2, Z3, Z4 and Z5 strains have been deposited on Genbank database with accession numbers: ON153202 to ON153211. And these sequences can be found here: https://www.ncbi.nlm.nih.gov/nuccore/?term=ON153202:ON153211[accn].
