# Correction to: Isolation and characterization of gut bacteria associated with the degradation of host-specific terpenoids in *Pagiophloeus tsushimanus* (Coleoptera: Curculionidae) larvae

**DOI:** 10.1093/jisesa/iead055

**Published:** 2023-06-30

This is a correction to: Heng Qiao and others, Isolation and characterization of gut bacteria associated with the degradation of host-specific terpenoids in *Pagiophloeus tsushimanus* (Coleoptera: Curculionidae) larvae, *Journal of Insect Science*, Volume 23, Issue 2, March 2023, 14, https://doi.org/10.1093/jisesa/iead019

In the originally published version of this manuscript, details were missing from the **Funding** statement. This should read additionally: “[...]the Shanghai Landscaping & City Appearance Administrative Bureau (Grant number G161206), Postgraduate Research & Practice Innovation Program of Jiangsu Province (Grant number KYCX23_1236), and National natural science foundation of China (Grant number 32271884).” instead of “[...]and the Shanghai Landscaping & City Appearance Administrative Bureau (Grant number G161206).”

This error has been corrected in the article.

