# Supplementary material for: Isolation and characterization of gut bacteria associated with the degradation of host-specific terpenoids in Pagiophloeus tsushimanus (Coleoptera: Curculionidae) larvae
Source: J Insect Sci. 2023 Apr 19;23(2):14. doi: 10.1093/jisesa/iead019 (PMC10114288; doi:10.1093/jisesa/iead019)
Supplement: iead019_suppl_Supplementary_Material [file iead019_suppl_supplementary_material.docx]

**Supplementary Materials:**

Figure S1. Sample collection for gas chromatography (GC)


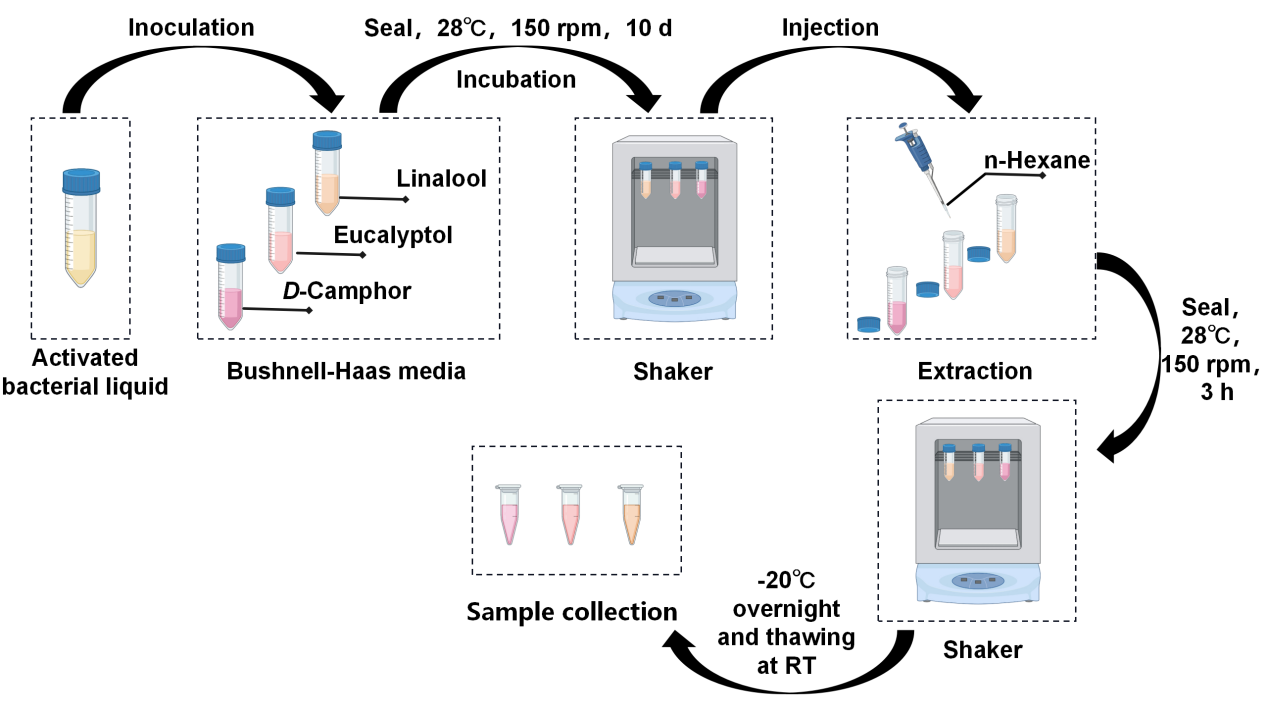


Figure S2. Standard curves of *D*-camphor (A), eucalyptol (B) and linalool (C).

| 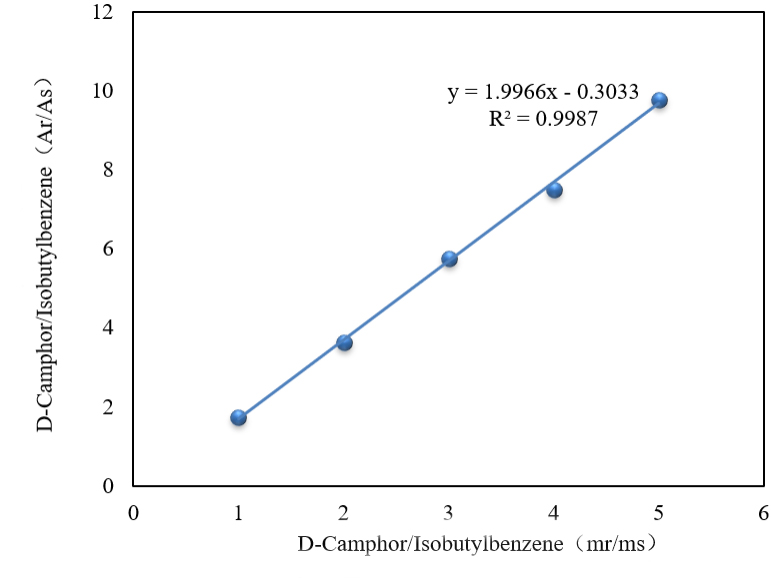 |
| --- |
| A |
| 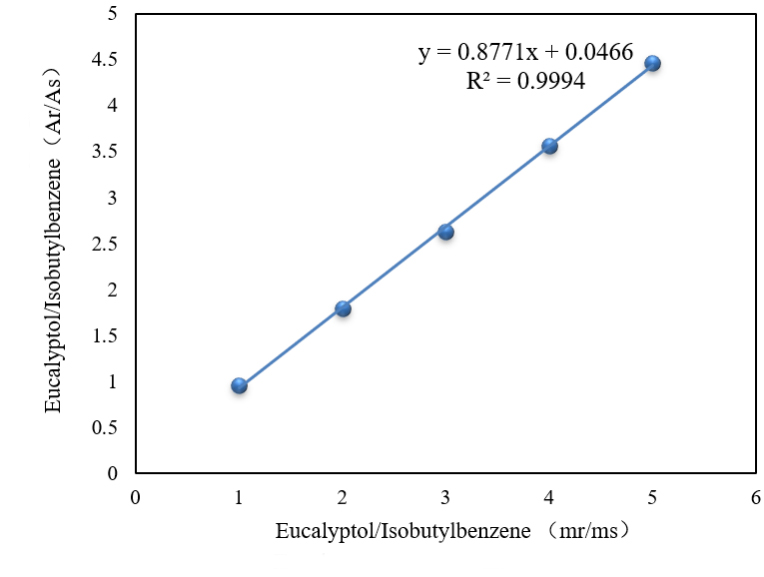 |
| B |
| 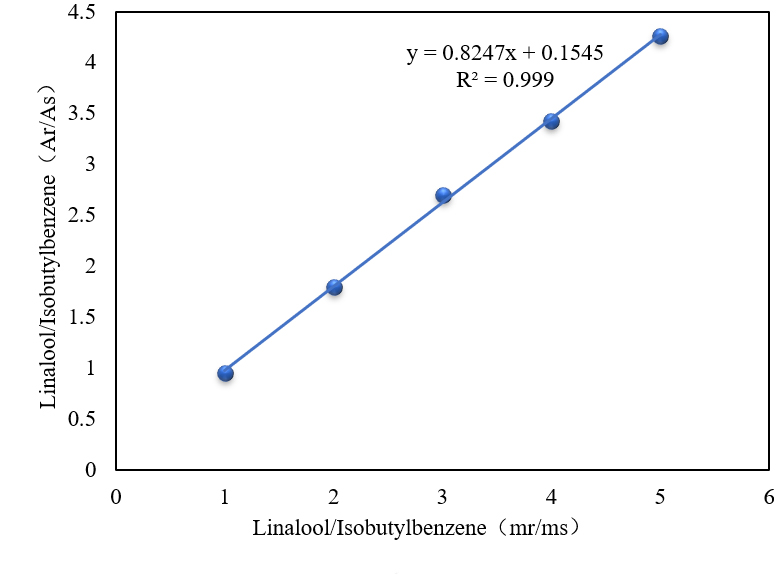 |
| C |
